# The association between FABP7 serum levels with survival and neurological complications in acetaminophen-induced acute liver failure: a nested case–control study

**DOI:** 10.1186/s13613-017-0323-0

**Published:** 2017-10-05

**Authors:** Constantine J. Karvellas, Jaime L. Speiser, Mélanie Tremblay, William M. Lee, Christopher F. Rose, For the US Acute Liver Failure Study Group

**Affiliations:** 1 Division of Gastroenterology (Liver Unit), Department of Critical Care Medicine, University of Alberta, 1-40 Zeidler Ledcor Building, Edmonton, AB T6G-2X8 Canada; 2 Department of Public Health Sciences, Medical University of South Carolina, Charleston, SC USA; 3 Hepato-Neuro Laboratory, CRCHUM, Université de Montréal, Montreal, Canada; 4 Division of Digestive and Liver Diseases, Department of Internal Medicine, University of Texas Southwestern Medical Center, Dallas, TX USA

**Keywords:** Liver-type fatty acid-binding protein, Multiorgan failure, Prognosis, ALFSG index

## Abstract

**Background:**

Acetaminophen (APAP)-induced acute liver failure (ALF) is associated with significant mortality due to intracranial hypertension (ICH), a result of cerebral edema (CE) and astrocyte swelling. Brain-type fatty acid-binding protein (FABP7) is a small (15 kDa) cytoplasmic protein abundantly expressed in astrocytes. The aim of this study was to determine whether serum FABP7 levels early (day 1) or late (days 3–5) level were associated with 21-day mortality and/or the presence of ICH/CE in APAP-ALF patients.

**Methods:**

Serum samples from 198 APAP-ALF patients (nested case–control study with 99 survivors and 99 non-survivors) were analyzed by ELISA methods and assessed with clinical data from the US Acute Liver Failure Study Group (ALFSG) Registry (1998–2014).

**Results:**

APAP-ALF survivors had significantly lower serum FABP7 levels on admission (147.9 vs. 316.5 ng/ml, *p* = 0.0002) and late (87.3 vs. 286.2 ng/ml, *p* < 0.0001) compared with non-survivors. However, a significant association between 21-day mortality and increased serum FABP7 early [log FABP7 odds ratio (OR) 1.16, *p* = 0.32] and late (log FABP7 ~ OR 1.34, *p* = 0.21) was not detected after adjusting for significant covariates (MELD, vasopressor use). Areas under the receiver-operating curve for early and late multivariable models were 0.760 and 0.892, respectively. In a second analysis, patients were grouped based on the presence (*n* = 46) or absence (*n* = 104) of ICH/CE. A significant difference in FABP7 levels between patients with or without ICH/CE at early (259.7 vs. 228.2 ng/ml, *p* = 0.61) and late (223.8 vs. 192.0 ng/ml, *p* = 0.19) time points was not identified.

**Conclusion:**

Serum FABP7 levels were significantly elevated at early and late time points in APAP-ALF non-survivors compared to survivors. However, significant differences in FABP7 levels by 21-day mortality were not ascertained after adjusting for significant covariates (reflecting severity of illness). Our study suggests that FABP7 may not discriminate between patients with or without intracranial complications.

**Electronic supplementary material:**

The online version of this article (doi:10.1186/s13613-017-0323-0) contains supplementary material, which is available to authorized users.

## Background

Acute liver failure (ALF) is defined by the occurrence of hepatic encephalopathy (HE) and hepatic synthetic dysfunction within 26 weeks of the first symptoms of liver disease [1]. Severe coagulopathy, encephalopathy and hemodynamic instability contribute to a picture of multiorgan failure. Currently, the most common cause of ALF in North America is acetaminophen (APAP) [2]. Particularly in APAP-induced ALF, cerebral edema (CE) and intracranial hypertension (ICH) are major causes of morbidity and mortality [3]. The pathogenesis for ICH and CE in ALF is not fully understood, but astrocyte swelling causing cellular dysfunction as well as increased cerebral blood flow is believed to be implicated [4]. The degree of hyperammonemia has been demonstrated to be associated with ICH [5]. Ammonia, as a gas (NH_3_) and ion (NH_4_^+^), freely crosses the blood–brain barrier and is primarily removed by glutamine synthetase, an enzyme solely found in astrocytes within the brain [6]. Glutamine synthetase catalyzes the amidation of glutamate to glutamine that subsequently leads to hyperosmotic changes and astrocyte swelling. However, studies have shown that hyperammonemia alone does not predict ICH [7].

Given the challenges presented in managing critically ill ALF patients with potential CE/ICH including the consideration for liver transplant (LT), the development of a noninvasive biomarker with the potential to predict ICH would be of great value, especially given the significant bleeding risks of invasive intracranial pressure monitoring in these coagulopathic patients [8].

Fatty acid-binding proteins (FABP) are small (15 kDa) cytoplasmic proteins that are abundantly expressed in tissues with active fatty acid metabolism, such as brain and liver. The primary function of FABPs is the intracellular transport of long-chain fatty acids [9]. The cellular expression of FABPs is responsive to changes in lipid metabolism, which can be induced during pathophysiological conditions, such as ischemia/inflammation or pharmacological stimuli [10]. Brain-type FABP (FABP7) is solely expressed in brain, exclusively in astrocytes [11]. Previous investigations have shown serum levels of FABP7 to be elevated in patients with various neurological diseases including stroke [12] and dementia [13]. While our group recently demonstrated the prognostic value of serum levels of liver-type FABP (FABP1) in ALF, to date FABP7 as a biomarker for the risk of ICH in ALF has not been investigated [14].

This nested case–control study of randomly selected samples from prospectively enrolled patients from the US Acute Liver Failure Study Group (ALFSG) registry aimed to examine levels of FABP7 in APAP-ALF patients. Specifically, our primary objectives were to test the following hypotheses

- Higher FABP7 serum levels are significantly associated with 21-day transplant-free mortality (in the absence of transplant) after adjusting for other significant covariates (Analysis 1).
- Elevated serum levels of FABP7 in APAP-ALF are significantly associated with ICH/CE after adjusting for other significant covariates (Analysis 2).

## Methods

This study is a nested case–control study of prospectively collected data and biosamples of 198 patients enrolled in the US ALFSG registry/biorepository and is outlined in detail in Additional file 1: Figure S1. Between January 1998 and December 2014, 1027 APAP-ALF patients were enrolled in the registry from which 704 patients were alive at day 21 in the absence of LT. We identified 124 survivors with early and late serum samples from which 99 were randomly selected for analysis. Of 224 patients who died in the absence of LT, 87 patients with early and late samples were also included in this analysis. A further 12 patients with exclusively an early sample (of a possible 92) were randomly selected for inclusion in this analysis. Personnel not involved in the analysis of the samples or statistical analysis for the paper performed random selection of patients. All enrolling centers were tertiary academic centers, and all but one were LT centers. The authors’ Institutional Review Board (IRB)/Health research ethics boards of all enrolling US ALFSG sites have approved all research, and all clinical investigation has been conducted according to the principles expressed in the 1975 Declaration of Helsinki. Given patients were unable to provide written consent (critical illness, HE), written assent was obtained from the next of kin from each patient. Each center implemented monitoring and therapeutic interventions according to institutional standards of care. Reporting of the analysis of this study followed the STROBE Guidelines for reporting case–control studies [15]. Consistent with ALFSG studies [16], the primary outcome (Analysis 1) was 21-day LT-free survival (no patients included in the analysis received LT). Secondary outcome (Analysis 2) was the development of ICH/CE.

### Participants

*Inclusion criteria* were: (1) evidence of ALF according to the enrollment criteria for the ALFSG (see operational definitions); (2) age ≥ 18 years; (3) HE during the first seven days of study admission (West Haven Criteria) [17]; and (4) patients within the ALFSG registry with primary diagnoses of APAP determined by the site investigator. *Exclusion criteria* were: (1) cirrhosis/acute-on chronic liver failure; (2) patients without a primary diagnosis of APAP; and (3) patients who received a LT. Serum samples were analyzed on study admission (early; day 1) and late (either day 3, 4 or 5) where available. Patients who received a LT *were excluded from our study* because listing for transplant is a clinical decision, which is not standardized among ALFSG sites. A further 51 healthy controls were analyzed (University of Alberta) for FABP7 only.

### Operational definitions

For the purposes of this study, ALF was defined as INR ≥ 1.5 and HE within the first 26 weeks of liver disease in a patient with an acute hepatic insult [18]. HE coma grade was defined by the West Haven Criteria (simplified) as follows: grade 1 ~ any alteration in mentation, grade 2 being somnolent or obtunded but easily rousable or presence of asterixis, grade 3 being rousable with difficulty, and grade 4 being unresponsive to deep pain [17]. In this study, we defined ‘low coma grade’ as grade 1 or 2 and ‘high coma grade’ as grade 3 or 4. The KCC [19] predicts poor outcome (death/transplant) if: (a) pH is less than 7.3 or (b) if INR is greater than 6.5, creatinine is greater than 3.4 mg/dl, and coma grade is high (3 or 4). The model for end-stage liver disease (MELD) is defined as 10*(0.957*log(4) + 0.378*log(bilirubin) + 1.12*log(INR)) for dialyzed patients and 10*(0.957*log(creatinine) + 0.378*log(bilirubin) + 1.12*log(INR)) for patients not dialyzed [20].

### Laboratory Assays of FABP7

FABP7 was measured in serum samples with a solid-phase enzyme-linked immunosorbent assay (ELISA) following manufacturer’s instructions (Biomatik, USA). Briefly, samples were incubated 2 h on a monoclonal anti-FABP7 pre-coated plate. A specific FABP7 biotin-conjugated polyclonal antibody solution was added for 2 h. After washing plates, avidin conjugated to horseradish peroxidase was added for 30 min. Finally, substrate tetramethylbenzidine was added for 15 min. Reactions were stopped by addition of sulfuric acid, and absorbance was read at 450 nm. Standard curve ranges from 0.47 to 30 ng/ml. Samples were performed in duplicate and accepted valid with a variation coefficient less than 25%.

### Statistical methods aim one: FABP7 and 21-day survival in APAP-ALF

For differences between outcome groups (APAP-ALF survivors, *n* = 99, APAP-ALF non-survivors, *n* = 99), categorical variables were compared using the Chi-squared test or Fisher’s exact test (if *n* < 10 in any cell of the two-by-two table). FABP7 was treated as a continuous variable. Continuous variables were reported as medians with interquartile range (IQR) and compared using the Wilcoxon rank-sum test. Survival was defined as the dichotomous outcome, alive or dead at 21 days after enrollment into the registry (no patients received a LT in this analysis). A two-sided *p* value of < 0.05 was considered statistically significant for all comparisons (Additional file 2).

In order to control for variables that may confound the effect of FABP7 on 21-day mortality, logistic regression analysis was performed [21]. Aside from FABP7, covariates considered in multivariable modeling included MELD, lactate, vasopressors use, RRT, MV and high coma grade. Separate multivariable (logistic) regression models were derived for FABP7 early (day 1) and late (days 3–5) by including variables, which were significant on univariate analysis and performing backward elimination with a p value threshold of 0.05.

### Statistical methods aim two: FABP7 and ICH in APAP-ALF

In this secondary analysis, the outcome of interest examined was intracranial hypertension (ICH) either based on (a) intracranial pressure monitoring with ICP > 25 mm Hg or based on (b) computed tomography (CT) imaging of the brain. CT evidence of cerebral edema was defined as a hypodense signal, effacement of the gray white matter junction, loss of differentiation of the lenticular nucleus and decreased visualization of the sulci, insula and cisterns [22]. Out of the 150 APAP-ALF patients where data were available to determine the presence or absence of ICH, 46 deceased patients had evidence of ICH based on these criteria. Statistical methods for this analysis will be similar to the first analysis except the primary outcome (ICH). Multivariable logistic regression analysis (as described above) was performed [21] to assess independent variables associated with ICH including FABP7. The pre-specified prognostic variables were based on previous publications [5] included at admission into the registry; age, lactate value, MELD [20] score (admission) and other variables with statistical significance on univariable analysis. Model performance for both Aim 1 and Aim 2 was assessed using area under the receiver-operating curve (AUROC) and the Hosmer–Lemeshow test for goodness of fit. SAS software version 9.3 was used for univariate comparisons and multivariable logistic regression modeling.

## Results

### Analysis one: comparative analysis of 198 APAP-ALF patients

Demographic and clinical outcomes stratified by mortality (alive at day 21, *n* = 99; deceased, *n* = 99) are listed in Table 1. No patients in this analysis received LT. Comparing APAP-ALF survivors and non-survivors at day 21, survivors required significantly less organ support during the 7 days of inpatient study (MV 65 vs. 93%; vasopressors 12 vs. 70%; RRT 27 vs. 45%; *p* < 0.008 for all). Survivors were less likely to achieve high (3 or 4) HE coma grade (62 vs. 93%, *p* < 0.0001) and less likely to receive mannitol for intracranial hypertension (22 vs. 46%, *p* = 0.0003). APAP-ALF survivors were less likely to have complications during the first 7 days of study including seizures (3 vs. 21%, *p* < 0.0001), arrhythmias (25 vs. 38%, *p* = 0.047) or gastrointestinal bleeding (8 vs. 19%, *p* = 0.037). On admission, 7% of APAP-ALF survivors and 16% of non-survivors met KCC (*p* = 0.13). Among the 99 APAP-ALF non-survivors, the most common causes of death reported were multiorgan failure (53%) and neurological complications (38%). Cause of death was unknown in 9% of cases.

**Table 1:** Demographic, clinical and biochemical parameters in 198 APAP-ALF patients by outcome

	APAP alive day 21 (*n* = 99)		APAP dead day 21 (*n* = 99)		*p* value
	N	Number (%) or median (IQR)	N	Number (%) or median (IQR)	
Age	99	35 (28–43)	99	40 (30–48)	0.084
Sex (female)	99	75 (76%)	99	72 (73%)	0.63
Race					0.23
White	99	83 (84%)	99	79 (80%)	
African-American	99	8 (8%)	99	15 (15%)	
Other	99	8 (8%)	99	5 (5%)	
Organ support (days 1–7)					
Mechanical ventilation	99	64 (65%)	99	93 (93%)	< 0.0001
Vasopressors	99	12 (12%)	99	69 (70%)	< 0.0001
Renal replacement therapy	99	27 (27%)	99	45 (45%)	0.0078
KCC	87	7 (7%)	87	16 (16%)	0.13
Coma grade 3/4 (worst days 1–7)	99	61 (62%)	98	91 (93%)	< 0.0001
ICP-directed therapies (days 1–7)					
ICP monitor	99	12 (12%)	99	21 (21%)	0.086
Mannitol	99	22 (22%)	99	46 (46%)	0.0003
Hypertonic saline	99	11 (11%)	99	14 (14%)	0.52
Barbiturates	99	9 (9%)	99	20 (20%)	0.043
Hypothermia	99	17 (17%)	99	14 (14%)	0.56
Sedatives	99	70 (71%)	99	88 (89%)	0.0014
Blood products (days 1–7)					
Red blood cells	99	34 (34%)	99	50 (51%)	0.021
Fresh-frozen plasma	99	50 (51%)	99	76 (77%)	0.0001
Recombinant VIIA	99	3 (3%)	99	5 (5%)	0.72
Platelets	99	17 (17%)	99	36 (36%)	0.0023
ICU complications (days 1–7)					
Seizures	99	3 (3%)	99	21 (21%)	< 0.0001
Arrhythmias	99	25 (25%)	99	38 (38%)	0.047
GI bleeding	99	8 (8%)	99	19 (19%)	0.037
ARDS	99	0 (0%)	99	3 (3%)	0.25
CT (cerebral edema)	55	7 (13%)	72	32 (44%)	< 0.001
Abnormal CXR	99	88 (89%)	99	83 (84%)	0.30
Bacteremia/blood stream infection	99	7 (7%)	99	10 (10%)	0.61
Cause of death					
Multiorgan failure			99	52 (53%)	
Cerebral edema			99	38 (38%)	
Unknown			99	9 (9%)	

*N* frequency, *IQR* interquartile range, *ARDS* acute respiratory syndrome, *CT* computed tomography, *CXR* chest x-ray

### Clinical parameters in 198 APAP-ALF patients: admission (early)

Comparisons of clinical parameters on study admission are listed in Table 2. APAP-ALF survivors demonstrated significantly lower MELD scores (23 vs. 29, *p* < 0.0001) than non-survivors on admission. Survivors were significantly less likely to be on organ support (MV 58 vs. 80%, *p* = 0.0007; vasopressors, 9 vs. 42%, *p* < 0.0001) or achieve high HE grade (57 vs. 71%, *p* = 0.034) on admission.

**Table 2:** Demographic, clinical and biochemical parameters in 198 APAP-ALF patients by outcome (admission)

Early (admission)	APAP alive day 21 (*n* = 99)		APAP dead day 21 (*n* = 99)		*p* value
	N	Number (%) or median (IQR)	N	Number (%) or median (IQR)	
Biochemistry					
Hemoglobin (g/dl)	99	10.4 (9.2–12.5)	97	10.9 (9.5–12.2)	0.52
White blood count (10^9^/l)	98	8.6 (6.4–11.2)	97	10.9 (7.3–17.5)	0.0008
Platelet count (10^9^/l)	98	132.5 (90.0–195.0)	97	110.0 (67.0–160.0)	0.0045
INR	99	2.7 (1.8–4.1)	96	3.4 (2.3–4.8)	0.0023
ALT (IU/l)	98	3380 (1949–6576)	99	3235 (1483–5716)	0.37
Bilirubin (mg/dl)	98	4.1 (2.5–5.6)	99	5.0 (3.6–7.8)	< 0.0001
pH	88	7.4 (7.4–7.5)	88	7.4 (7.3–7.5)	0.22
Ammonia (venous) (μmol/l)	51	92 (73–140)	32	139 (72–205)	0.068
Creatinine (mg/dl)	98	1.4 (0.8–3.0)	98	2.6 (1.2–3.8)	0.0007
Lactate (mmol/l)	71	2.8 (1.7–5.5)	68	7.0 (4.8–11.8)	< 0.0001
Phosphate (mg/dl)	88	2.3 (1.7–3.4)	76	3.2 (2.1–4.5)	0.0061
MELD	98	23.4 (12.7–27.7)	96	29.1 (23.8–34.5)	< 0.0001
High coma grade (3 or 4)	99	56 (57%)	97	69 (71%)	0.034
Organ support					
Mechanical ventilation	99	57(58%)	99	79 (80%)	0.0007
Vasopressors	99	9 (9%)	99	42 (42%)	< 0.0001
Renal replacement therapy	99	19 (19%)	99	24 (24%)	0.39
FABP7 (ng/ml)	99	147.9 (66.6–296.2)	99	316.5 (119.8–562.2)	0.0002

Late (*n* = 186)	APAP alive day 21 (*n* = 99)		APAP dead day 21 (*n* = 87)		*p* value
Late (days 3–5)	N	Number (%) or median (IQR)	N	Number (%) or median (IQR)	
Biochemistry					
Hemoglobin (g/dl)	94	9.9 (9.0–11.1)	81	10.2 (9.2–10.9)	0.63
White blood count (10^9^/L)	94	8.1 (5.9–11.6)	81	11.3 (7.1–15.0)	0.0029
Platelet count (10^9^/L)	95	111.0 (68.0–153.0)	81	66.0 (47.0–100.0)	< 0.0001
INR	94	1.5 (1.3–1.8)	75	2.5 (1.8–4.4)	< 0.0001
ALT (IU/L)	94	1172 (612–2007)	78	938 (383–1995)	0.31
Bilirubin (mg/dl)	92	5.5 (2.7–8.2)	78	9.8 (6.7–13.7)	< 0.0001
pH	55	7.4 (7.4–7.5)	76	7.4 (7.3–7.5)	0.042
Ammonia (venous) (μmol/L)	25	62 (44–84)	17	119 (78–133)	0.0038
Creatinine (mg/dl)	94	1.2 (0.7–2.5)	82	2.4 (1.3–4.0)	< 0.0001
Lactate (mmol/L)	31	1.7 (1.0–2.2)	41	3.8 (2.6–6.7)	< 0.0001
Phosphate (mg/dl)	73	2.8 (2.3–3.6)	35	3.3 (2.5–4.5)	0.054
PO_2_/FiO_2_ ratio	48	3.3 (2.1–4.5)	71	2.5 (1.4–3.9)	0.0087
MELD	87	14.2 (5.4–24.6)	73	29.7 (23.5–35.4)	< 0.0001
High coma grade (3 or 4)^a^	59	35 (59%)	82	72 (88%)	< 0.0001
Organ support					
Mechanical ventilation	99	49 (49%)	88	74 (85%)	< 0.0001
Vasopressors	99	5 (5%)	88	45 (52%)	< 0.0001
Renal replacement therapy	99	10 (19%)	88	27 (31%)	0.062
FABP7 (ng/ml)	99	87.3 (48.0–261.5)	87	286.2 (146.7–536.9)	< 0.0001

*N* frequency, *IQR* interquartile range, *INR* international normalized ratio, *AST* aspartate aminotransferase, *ALT* alanine aminotransferase, *MELD* model for end-stage liver disease

^a^Hepatic encephalopathy grade according to West Haven criteria

**Fig. 1:**
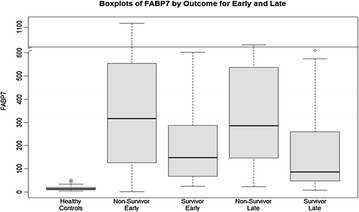
Serum levels of FABP7 (ng/ml) in healthy controls, non-survivors (early ~ admission), survivors (early), non-survivors (late ~ days 3–5), survivors (late)

FABP7 levels at admission (early) are listed in Table 2 and graphically shown in Fig. 1. APAP-ALF survivors had significantly lower admission serum FABP7 levels (147.9 vs. 316.5 ng/ml) compared with non-survivors (*p* = 0.0002). In comparison, 52 healthy controls had median serum levels of 13.5 (8.7–20.2) ng/ml.

### Clinical parameters in 186 APAP-ALF patients: days 3–5 (late)

Comparisons of clinical parameters on *days 3*–*5 (late)* are listed in Table 2. Of the 99 APAP-ALF non-survivors, samples of late time points were available in 87 patients as 12 died before days 3–5. APAP-ALF survivors (*n* = 99) were significantly less likely to be on MV (49 vs. 85%, p < 0.0001) and vasopressors, (5 vs. 52%, p < 0.0001) or achieve high HE grade (59 vs. 88%, p < 0.0001) than non-survivors.

Late (days 3–5) FABP7 are listed in Table 2 and graphically shown in Fig. 1. APAP-ALF survivors had significantly lower late serum FABP7 levels (87.3 vs. 286.2 ng/ml) compared with non-survivors (*p* < 0.0001). FABP7 levels were significantly higher in all ALF patients (survivors and non-survivors) compared to healthy controls for both early and late time points (*p* < 0.0001).

### Multivariable analysis: associations with 21-day mortality

In order to adjust for covariates, multivariable logistic regression for 198 APAP-ALF patients to determine associations (adjusted) with 21-day mortality was performed (Table 3). Two models were derived: one on admission (early) and one at days 3–5 (late). Values of serum FABP7 were transformed to their natural logarithm (log FABP1) to comply with the linearity assumption in logistic regression.

**Table 3:** Early (day 1) and late (days 3–5) predictors of 21-day mortality in 198 APAP-ALF patients

Early	Unadjusted				Multivariable model (*N* = 194), AUROC = 0.766			
	N	OR	95% OR CI	*p* value	Included in Model	OR	95% CI	*p* value
FABP7	198	1.001	(1.000, 1.002)	0.0078	Yes	1.001	(1.000, 1.001)	0.18
MELD	194	1.083	(1.047, 1.119)	< 0.0001	Yes	1.056	(1.020, 1.093)	0.0021
Lactate	132	1.205	(1.095, 1.327)	0.0001	No			
Vasopressors	198	7.368	(3.335, 16.287)	< 0.0001	Yes	4.138	(1.769, 9.677)	0.0011
Renal replacement therapy	198	1.347	(0.683, 2.658)	0.390	No			
Mechanical ventilation	198	2.910	(1.547, 5.475)	0.0009	No			
High coma grade (3 or 4)	196	1.892	(1.047, 3.421)	0.0348	No			

Late (days 3–5)	Unadjusted				Multivariable model (*N* = 160), AUROC = 0.891			
	N	OR	95% OR CI	*p* value	Included in model	OR	95% CI	*p* value
FABP7	186	1.003	(1.001, 1.004)	0.0001	Yes	1.001	(0.999, 1.003)	0.40
MELD	160	1.115	(1.075, 1.157)	< 0.0001	Yes	1.084	(1.038, 1.132)	0.0003
Lactate	71	6.908	(2.592, 18.406)	0.0001	No			
Vasopressors	186	20.143	(7.462, 54.370)	< 0.0001	Yes	20.419	(6.221, 67.021)	<0.0001
Renal replacement therapy	186	1.895	(0.964, 3.724)	0.0638	No			
Mechanical ventilation	186	5.808	(2.859, 11.802)	< 0.0001	No			
High coma grade (3 or 4)	141	4.936	(2.129, 11.446)	0.0002	No			

Early: lactate (*p* = 0.52), high coma grade (*p* = 0.46), and mechanical ventilator (*p* = 0.084) were not significant on multivariable analysis so not included in the final early model

Late: mechanical ventilation (*p* = 0.69) and high coma grade (*p* = 0.53) were not significant on multivariable analysis so not included in the final late model. Lactate was not included due to missing data

### Early (admission) model

FABP7 was not associated with 21-day mortality [odds ratio OR 1.001 per increment, 95% CI (1.000, 1.001), *p* = 0.18] after adjusting for significant covariates including MELD [OR 1.056 (1.020, 1.093) per increment, *p* = 0.0021] and requirement for vasopressors [OR 4.14 (1.77, 9.07), *p* = 0.0011]. This early model demonstrated AUROC of 0.766.

### Late (days 3–5) model

FABP7 was not associated with 21-day mortality [OR 1.001 (0.999, 1.003) per increment, *p* = 0.40] after adjusting for significant covariates including MELD [OR 1.084 (1.038, 1.132) per increment, *p* = 0.0003] and requirement for vasopressors [OR 20.42 (6.22, 67.02), *p* < 0.0001]. This late model demonstrated AUROC of 0.891.

### Analysis two: comparative analysis of 150 APAP-ALF patients

Demographic and clinical outcomes of 150 patients stratified by the presence (*n* = 46) and absence (*n* = 104) of ICH/CE based on review of subject data (ICP measurements, CT brain, cause of death) are shown in Additional file 3: Table S1. (In 48 patients, the presence or absence of ICH/CE could not be determined.) There were no significant differences in age (36 vs. 39, p = 0.11) or gender (female 74 vs. 69%, *p* = 0.56). During the 7 days of inpatient study, APAP-ALF patients with ICH/CE had higher requirements for ventilation (MV 100 vs. 75%, *p* < 0.0001) and were more likely to achieve high (3 or 4) HE coma grade (100 vs. 72%, *p* < 0.0001). APAP-ALF patients with evidence of ICH/CE were less likely to be alive at day 21 (17 vs. 41%, *p* = 0.0049) but were more likely to be listed for LT (33 vs. 13%, *p* = 0.0062).

### Clinical parameters in 150 APAP-ALF patients: admission (early)

**Fig. 2:**
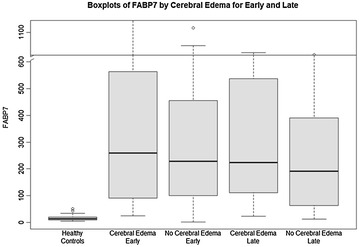
Serum levels of FABP7 (ng/ml) in healthy controls, cerebral edema (early ~ admission), no cerebral edema (early), cerebral edema (late ~ days 3–5), no cerebral edema (late)

Comparisons of clinical parameters on study admission are shown in Additional file 4: Table S2. APAP-ALF patients with ICH/CE had significantly higher serum INR (3.6 vs. 2.9) compared to patients without ICH/CE (*p* = 0.024). On study admission, patients who went on to develop ICH/CE were significantly more likely to be on mechanical ventilation (MV 85 vs. 65%, p = 0.019) and achieve high HE grade (76 vs. 59%, *p* = 0.043). Admission (early) levels of FABP7 are listed in Additional file 4: Table S2 and graphically shown in Fig. 2. There were no significant differences in FABP7 levels on admission between APAP-ALF patients with or without ICH/CE (259.7 vs. 228.2 ng/ml, *p* = 0.61).

### Clinical parameters in 186 APAP-ALF patients: days 3–5 (late)

Comparisons of clinical parameters on *days 3*–*5 (late)* are shown in Additional file 4: Table S2. Patients who developed ICH/CE were significantly more likely to be on mechanical ventilation (MV 95 vs. 63%, *p* < 0.0001) and achieve higher grades of HE (100 vs. 75%, *p* = 0.0004). Days 3–5 (late) levels of FABP7 are shown in Additional file 4: Table S2 and graphically in Fig. 2. There were no significant differences in late FABP7 levels between APAP-ALF patients with or without ICH/CE (223.8 vs. 192.0 ng/ml, *p* = 0.19).

### Multivariable analysis: associations with 21-day mortality

Multivariable logistic regression for 198 APAP-ALF to determine associations (adjusted) with the development of ICH/CE was performed (Table 4). Two models were derived; one on admission (early) and one at days 3–5 (late). Values of serum FABP7 were transformed to their natural logarithm (log FABP1) to comply with the linearity assumption in logistic regression.

**Table 4:** Early (admission) and late (days 3–5) predictors of cerebral edema in 150 APAP-ALF patients

Early	Unadjusted				Multivariable model (*N* = 148), AUROC = 0.590			
	N	OR	95% OR CI	*p* value	Included in model	OR	95% CI	*p* value
FABP7	150	1.000	(1.000, 1.001)	0.58	Yes	1.000	(1.000, 1.001)	0.65
MELD	146	1.036	(0.997, 1.078)	0.072	No			
Lactate	100	1.000	(0.997, 1.002)	0.80	No			
Vasopressors	150	0.777	(0.363, 1.660)	0.51	No			
Renal replacement therapy	150	1.313	(0.598, 2.886)	0.50	No			
Mechanical ventilation	150	2.950	(1.199, 7.257)	0.019	Yes	2.880	(1.166, 7.111)	0.022
High coma grade (3 or 4)	148	2.227	(1.017, 4.878)	0.045	No			

Late (days 3–5)	Unadjusted				Multivariable model (*N* = 113), AUROC = 0.641			
	N	OR	95% OR CI	*p* value	Included in model	OR	95% CI	*p* value
FABP7	138	1.000	(0.999, 1.001)	0.96	Yes	1.000	(0.999, 1.001)	0.57
MELD	118	1.030	(0.994, 1.066)	0.10	No			
Lactate	53	1.031	(0.979, 1.086)	0.25	No			
Vasopressors	138	2.043	(0.947, 4.408)	0.069	No			
Renal replacement therapy	138	1.756	(0.789, 3.909)	0.17	No			
Mechanical ventilation	138	10.115	(2.300, 44.491)	0.0022	No			
High coma grade (3 or 4)*	113	24.324	(1.326, 446.243)	0.0316	Yes	25.759	(1.404, 472.46)	0.029

Early: high coma grade (*p* = 0.69) was not significant on multivariable analysis so not included in the final early model

Late: mechanical ventilation (*p* = 0.65) was not significant on multivariable analysis so not included in the final late model

*Statistically significant on multivariable analysis

### Early (admission) model

FABP7 was not associated with the development of ICH/CE [OR 1.000 per increment, 95% CI (1.000, 1.001), *p* = 0.65] after adjusting for the only significant covariate, mechanical ventilation [OR 2.880 (1.166, 7.111), *p* = 0.022]. This early model demonstrated AUROC of 0.590.

### Late (days 3–5) model

FABP7 was not associated with the development of ICH/CE [OR 1.000 per increment, 95% CI (0.999, 1.001), *p* = 0.57] after adjusting for the only significant covariate, high hepatic coma grade [OR 25.76 (1.40, 472.5), *p* = 0.029]. This late model demonstrated AUROC of 0.641.

## Discussion

### Key results

In this nested case–control study, we report the first published analysis of FABP7 in a large series of 198 well-characterized APAP-ALF patients. Compared with survivors, serum FABP7 levels were significantly higher at serial time points (early and late) in APAP-ALF non-survivors. However, significant differences in FABP7 levels by 21-day mortality were not ascertained after adjusting for significant covariates reflecting severity of illness (MELD, vasopressor dependence). No differences in the FABP7 levels were detected for APAP-ALF patients with and without evidence of ICH/CE.

### Comparison with literature

In our study, ICH/CE was the cause of death in 39% of patients, similar to what has been previously reported [16]. ICH/CE arises due to astrocyte swelling, cerebral vasodilatation, dilated cerebral arterioles and altered cerebral blood flow [23, 24]. Furthermore, it has been shown that patients with signs of cerebral edema and ICH have increased cerebral blood flow compared to patients without brain edema [25, 26]. Given the bleeding risks associated with direct intracranial pressure monitoring [8], there is an unmet need for noninvasive markers of brain edema and ICH to help inform medical decisions. In the setting of ALF, astrocyte swelling/injury leads to astrocyte dysfunction and consequently impairs neuronal function leading to HE. However, in parallel, swollen astrocytes release small proteins, molecules and osmolytes in response to astrocyte hypertonicity to reduce swelling. In the past 20 years, several biochemical biomarkers have been investigated for the detection of cerebral injury, including protein S100b [27], neuron-specific enolase (NSE) [27] and glial fibrillary protein (GFAP) [9]. Studies by Strauss et al. [27], as well as Vaquero et al. [28], which included 35 and 54 ALF patients, respectively, concluded that S-100b was not a useful marker of neurological outcome in ALF. Furthermore, despite a consistent increase of S-100b in serum, levels did not correlate with severity of HE, development of brain herniation or outcome. In the same patients, Strauss et al. found that serum levels of NSE were higher in ALF patients with ICH than those who survived without ICH [27]. However, this univariate comparison did not adjust for significant confounding factors/covariates, an important limitation to the study.

Major components of the brain are lipids with brain cells having a high cell membrane/cytoplasm ratio. Cell membranes are formed of lipid bilayers consisting of saturated and unsaturated fatty acids, which can also be oxidized for generating ATP. The primary function of FABPs is to facilitate the transport of intracellular long-chain fatty acids. In the brain, FABP7 and heart FABP (FABP3) are expressed with FABP7 primarily found in astrocytes [29] and FABP3 in neurons [30]. With astrocyte swelling being a neuropathological landmark of ALF along with a hyperdynamic circulation frequently occurring in ALF leading to an increased myocardial demand, FABP3 may be confounded with myocardial injury. Therefore, FABP7 is more specific to brain injury in ALF.

FABP7 has physiological properties that render this protein advantageous as a prognostic biomarker in ALF: (i) it is abundantly present in astrocytes (between approximately 0.8 and 3.1 μg/g of brain tissue), (ii) it has a lower molecular mass (14 kDa); it is smaller than S-100b (21 kDa), enolase (47 kDa) and GFAP (50 kDa) with a much shorter plasma half-life (11 min) [31–33]. Smaller proteins such as FABP7 diffuse more rapidly (via transcytosis) than larger proteins though the interstitial space and cross the blood–brain barrier (BBB).

The release of cerebrovascular proteins into blood plasma is dependent on the status (breakdown) of the BBB, which in ALF is dependent on the underlying mechanisms of cerebral edema, cytotoxic and vasogenic [34]. Astrocyte swelling plays a definitive role in the development of cytotoxic brain edema. In cytotoxic edema, the BBB is intact in the presence of intracellular swelling [35], whereas in vasogenic edema there is breakdown of the BBB and water and plasma constituents accumulate in the extracellular space [36]. Although a complete breakdown of the blood–brain barrier is not evident in ALF, increased permeation to water and other small molecules such as ammonia has been demonstrated resulting from subtle alterations in the protein composition of paracellular tight junctions [37].

Despite elevated levels in ALF patients (survivors and non-survivors) in this analysis, FABP7 did not discriminate between patients who went on to develop significant signs of ICH/CE either on imaging, ICP measurements or at death. One explanation is that variability in BBB permeability during ICH/CE could impact the diffusion rate of FABP7 into the peripheral circulation. In this study, we speculate that heterogeneity in the permeability of the BBB in ALF patients likely impacted the discriminatory ability of serum FABP7 measurements and important neurological outcomes in ALF. While FABP7 in cerebrospinal fluid may be more discriminatory between patients with and without astrocyte swelling/injury, this would not be feasible as a noninvasive biomarker as it would require an interventricular drain.

## Limitations

The following limitations of this study warrant consideration. It is a nested case–control study, and as such the event rate of the primary outcome (21-day mortality) was 50%, higher than published in cohort series. Although patients were enrolled and samples were collected prospectively, analysis was performed retrospectively and therefore can comment on association and discrimination (between survivors and non-survivors) and not on the absolute risks of death and intracranial complications according to serum FABP7 levels. To account for potential confounding in the study design, we performed multivariable analysis to adjust for other significant covariates reflecting severity of illness (MELD, vasopressors, mechanical ventilation, hepatic coma grade). To avoid confounding related to LT since transplant listing decisions for APAP-ALF and the organ availability were not consistent between study centers (Simmons et al., ALFSG unpublished data), samples from patients who received a LT were not evaluated in this study. The case–control design of the study may have introduced selection bias, as the primary outcome of survival is automatically unbalanced within the clinical profile of the groups. However, in an attempt to reduce observation bias, data were collected prospectively and within this specific study design, researchers measuring FABP7 were blinded to the clinical and outcome data of patients at the time of patient selection and sample analysis. Finally, we acknowledge that determinations of ICH were done retrospectively using available data from death summaries: cranial imaging and ICP measurements (if a monitor was used), and this may have introduced further bias. The decision to order computed tomography of the brain and the use of ICP monitors were individual decisions made by the practicing clinician, and these were not standardized across the ALFSG registry and may have varied between centers. While renal function may have impacted serum FABP7 levels, we attempted to adjust for this by including renal function (MELD) in multivariable analysis. Nonetheless despite these limitations, we believe these results are robust as they include APAP-ALF cases from across 16 tertiary liver transplant centers comprising the US ALFSG and are the first report of FABP7 in acute liver failure.

## Conclusions

Brain FABP levels were elevated in APAP-ALF patients with significantly higher serum levels at early and late time points in APAP-ALF non-survivors. However, significant differences in FABP7 levels by 21-day mortality were not ascertained after adjusting for significant covariates reflecting severity of illness (MELD, vasopressor dependence). No differences in the FABP7 levels were detected for APAP-ALF patients with and without evidence of ICH/CE. FABP7 does not appear to discriminate between patients who did and did not have significant intracranial complications of APAP-ALF.

## Additional files

**Additional file 1. Figure S1.** APAP-ALF patients in ALFSG registry as of January 1, 2015.

**Additional file 2.** Receiver operator curve (ROC) for independent predictors of 21-day mortality in APAP-ALF patients.

**Additional file 3. Table S1.** Demographic and clinical parameters in 150 APAP-ALF patients stratified by cerebral edema.

**Additional file 4. Table S2.** Biochemical and organ support parameters early (admission) and late (days 3–5) stratified cerebral edema.

**Additional file 5.** STROBE Statement—Checklist of items that should be included in reports of *case*–*control studies*.

## Abbreviations

ALF: acute liver failure
ALFSG: Acute Liver Failure Study Group
APAP: acetaminophen
FABP7: brain-type fatty acid-binding protein
HE: hepatic encephalopathy
ICU: intensive care unit
INR: international normalized ratio
IQR: interquartile range
KCC: King’s College criteria
LT: liver transplantation
MELD: model for end-stage liver disease score
MV: mechanical ventilation
OR: odds ratio
RRT: renal replacement therapy
