# Supplementary material for: The association between FABP7 serum levels with survival and neurological complications in acetaminophen-induced acute liver failure: a nested case–control study
Source: Ann Intensive Care. 2017 Oct 5;7:99. doi: 10.1186/s13613-017-0323-0 (PMC5629189; doi:10.1186/s13613-017-0323-0)
Supplement: Supplementary file 1 — Additional file 1. Figure S1. APAP-ALF patients in ALFSG registry as of January 1, 2015. [file 13613_2017_323_MOESM1_ESM.pdf]

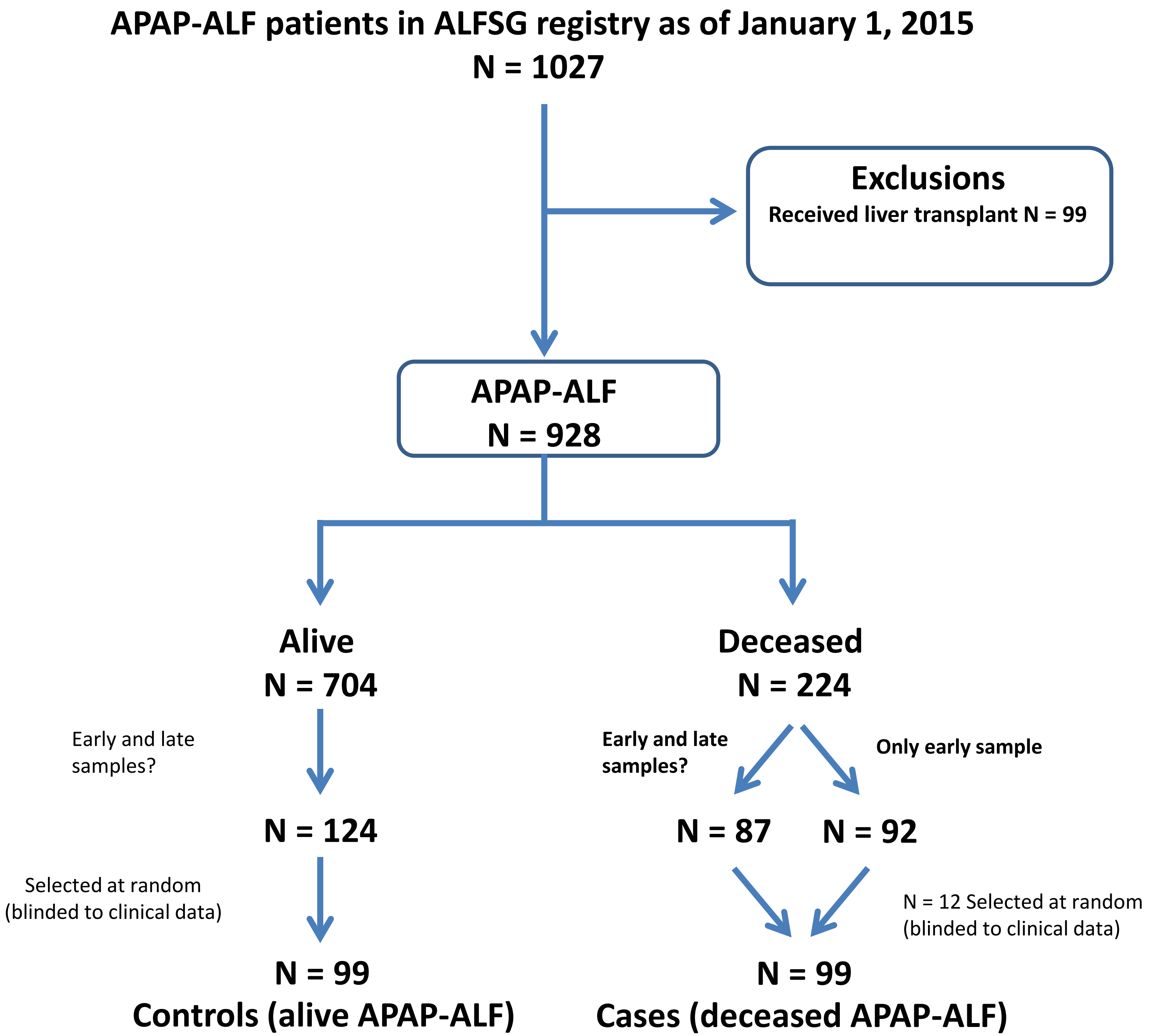
APAP-ALF patients in ALFSG registry as of January 1, 2015
N = 1027
Exclusions
Received liver transplant N = 99
APAP-ALF
N = 928
Alive
N = 704
Deceased
N = 224
Early and late samples?
N = 124
Early and late samples?
Only early sample
N = 87
N = 92
Selected at random (blinded to clinical data)
N = 12 Selected at random (blinded to clinical data)
N = 99
Controls (alive APAP-ALF)
N = 99
Cases (deceased APAP-ALF)
