# Supplementary material for: The association between FABP7 serum levels with survival and neurological complications in acetaminophen-induced acute liver failure: a nested case–control study
Source: Ann Intensive Care. 2017 Oct 5;7:99. doi: 10.1186/s13613-017-0323-0 (PMC5629189; doi:10.1186/s13613-017-0323-0)

**Supplementary Figure:** Receiver Operator Curve (ROC) for Independent Predictors of

21-day mortality in APAP-ALF patients

- Early model AUROC=0.766
- Late model AUROC=0.891


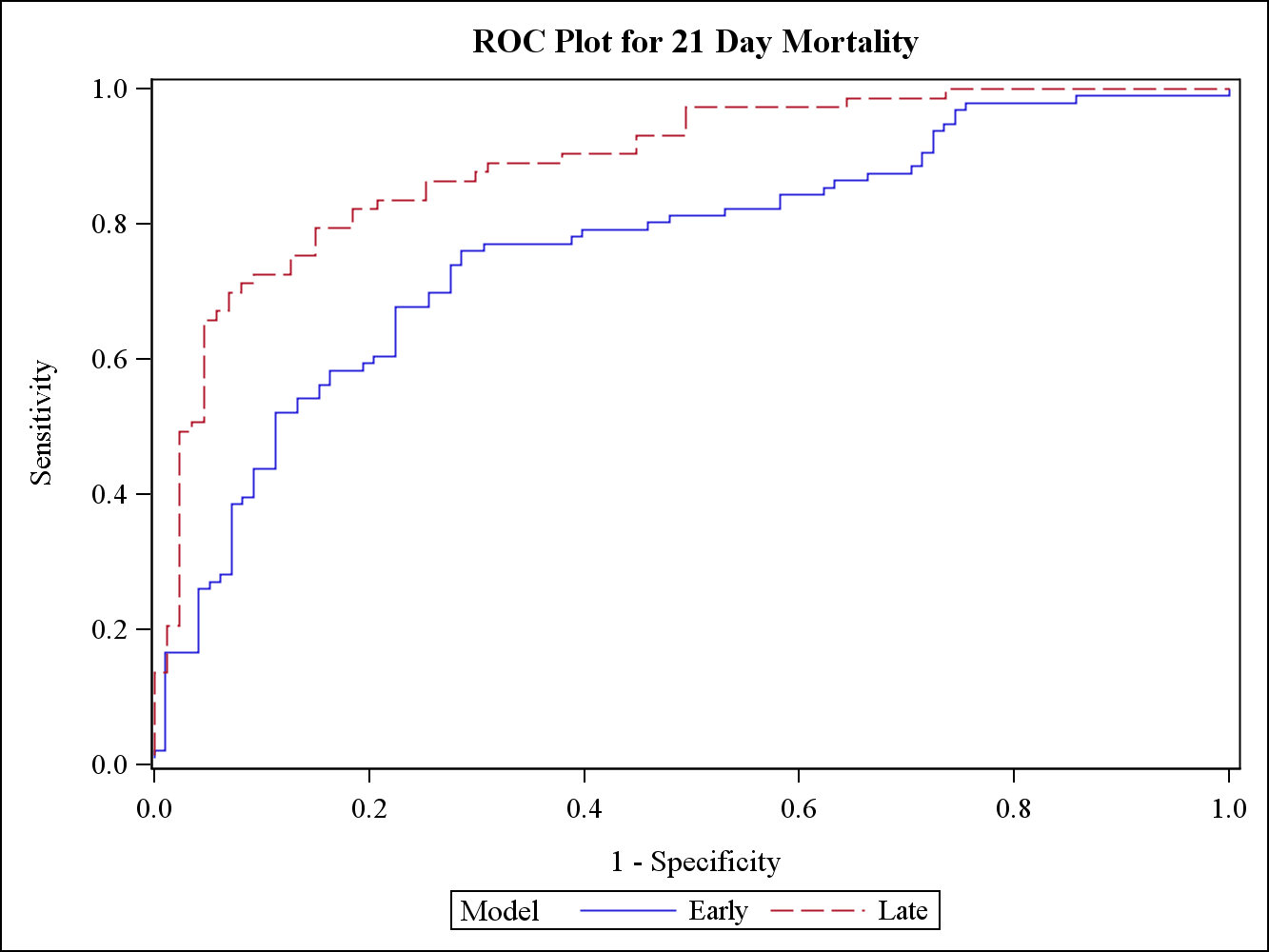


**Supplementary Figure:** Receiver Operator Curve (ROC) for Independent Predictors of

Cerebral edema in 150 APAP-ALF patients

- Early model AUROC=0.590
- Late model AUROC=0.641


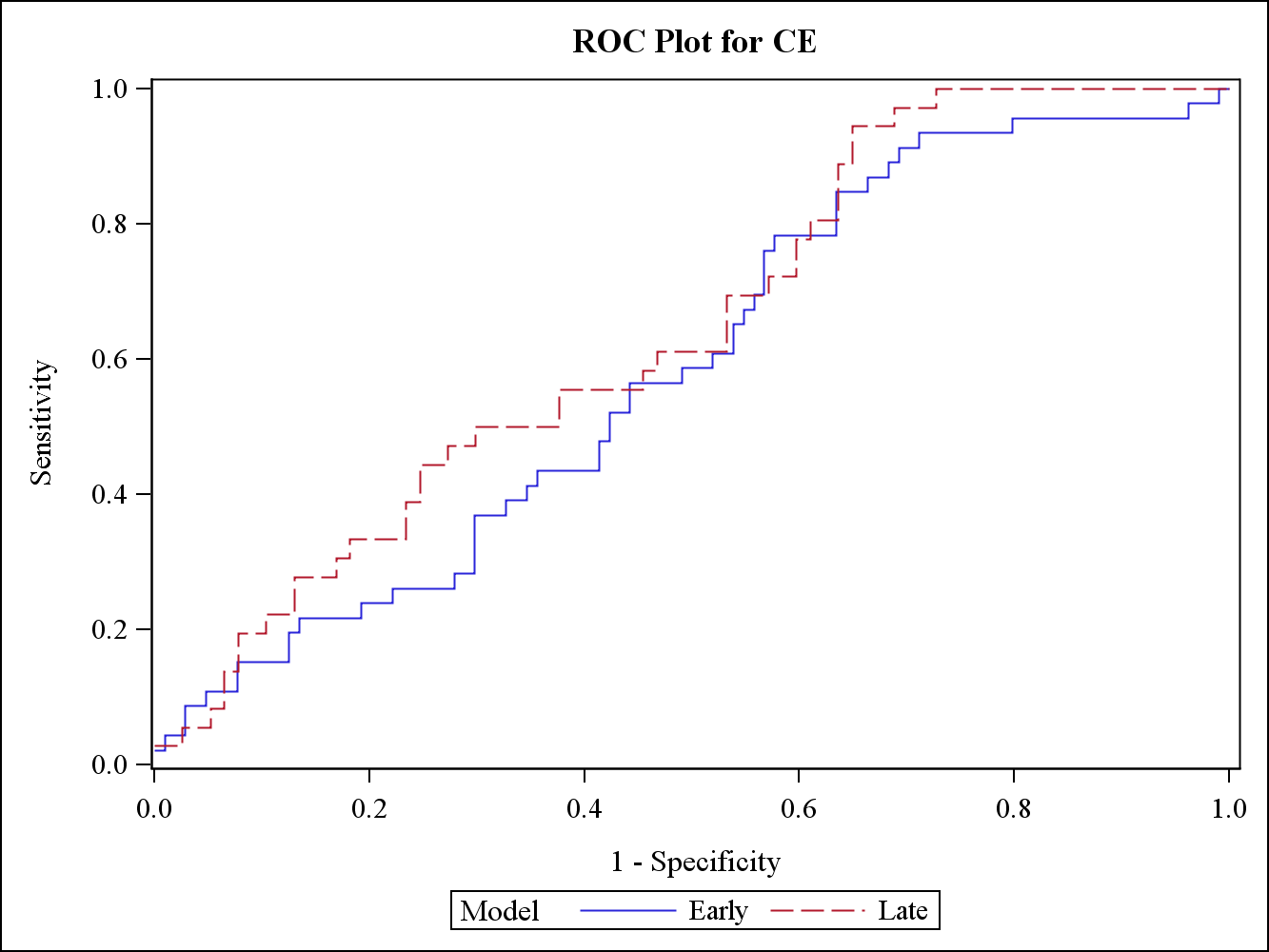

Supplement: Supplementary file 2 — Additional file 2. Receiver operator curve (ROC) for independent predictors of 21-day mortality in APAP-ALF patients. [file 13613_2017_323_MOESM2_ESM.docx]
