# Supplementary material for: The association between FABP7 serum levels with survival and neurological complications in acetaminophen-induced acute liver failure: a nested case–control study
Source: Ann Intensive Care. 2017 Oct 5;7:99. doi: 10.1186/s13613-017-0323-0 (PMC5629189; doi:10.1186/s13613-017-0323-0)
Supplement: Supplementary file 3 — Additional file 3. Table S1. Demographic and clinical parameters in 150 APAP-ALF patients stratified by cerebral edema. [file 13613_2017_323_MOESM3_ESM.docx]

**Table S1.** Demographic and Clinical Parameters in 150 APAP-ALF patients stratified by Cerebral Edema.

|  | **APAP Cerebral Edema**  **(n=46)** | | **APAP No Cerebral Edema**  **(n=104)** | |  |
| --- | --- | --- | --- | --- | --- |
|  | N | Number (%) or median (IQR) | N | Number (%) or median (IQR) | P value |
| **Age** | 46 | 36 (24-44) | 104 | 39 (30-48) | 0.11 |
| **Sex (female)** | 46 | 34 (74%) | 104 | 72 (69%) | 0.56 |
| **Race** |  |  |  |  | 0.36 |
| White | 46 | 40 (87%) | 104 | 81 (78%) |  |
| African-American | 46 | 3 (7%) | 104 | 16 (15%) |  |
| Other | 46 | 3 (7%) | 104 | 7 (7%) |  |
| **Organ support (days 1-7)** |  |  |  |  |  |
| Mechanical ventilation | 46 | 46 (100%) | 104 | 78 (75%) | <0.0001 |
| Vasopressors | 46 | 28 (61%) | 104 | 48 (46%) | 0.097 |
| Renal Replacement therapy | 46 | 21 (46%) | 104 | 40 (38%) | 0.41 |
| **KCC** | 41 | 5 (12%) | 89 | 16 (18%) | 0.92 |
| **Coma Grade 3/ 4 (Worst days 1-7)** | 46 | 46 (100%) | 103 | 74 (72%) | <0.0001 |
| **ICP directed therapies (days 1-7)** | 46 | 19 (41%) | 104 | 8 (8%) | <0.0001 |
| ICP Monitor | 46 | 37 (80%) | 104 | 24 (23%) | <0.0001 |
| Mannitol | 46 | 8 (17%) | 104 | 12 (12%) | 0.43 |
| Hypertonic saline | 46 | 11 (24%) | 104 | 11 (11%) | 0.033 |
| Barbiturates | 46 | 13 (28%) | 104 | 10 (10%) | 0.0060 |
| Hypothermia | 46 | 43 (93%) | 104 | 81 (78%) | 0.020 |
| Sedatives |  |  |  |  |  |
| **Blood products (days 1-7)** | 46 | 24 (52%) | 104 | 43 (41%) | 0.22 |
| Red Blood Cells | 46 | 37 (80%) | 104 | 67 (64%) | 0.056 |
| Fresh Frozen Plasma | 46 | 4 (9%) | 104 | 3 (3%) | 0.20 |
| Recombinant VIIA | 46 | 15 (33%) | 104 | 32 (31%) | 0.82 |
| Platelets |  |  |  |  |  |
| **ICU Complications (days 1-7)** | 46 | 12 (26%) | 104 | 11 (11%) | 0.015 |
| Seizures | 46 | 18 (39%) | 104 | 35 (34%) | 0.52 |
| Arrhythmias | 46 | 9 (20%) | 104 | 16 (15%) | 0.64 |
| GI bleeding | 46 | 0 (0%) | 104 | 3 (3%) | 0.55 |
| Abnormal CT | **44** | **39 (89%)** | **79** | **0 (0%)** | **< 0.001** |
| Abnormal CXR | 46 | 37 (80%) | 104 | 89 (86%) | 0.47 |
| Bacteremia/Blood stream infection | 46 | 2 (4%) | 104 | 13 (13%) | 0.15 |
| **Alive by Day 21** | 46 | 8 (17%) | 104 | 43 (41%) | 0.0049 |
| **Listed for transplant** | 46 | 15 (33%) | 104 | 14 (13%) | 0.0062 |

N: frequency. IQR: interquartile range.

ARDS: acute respiratory syndrome. CT: computed tomography. CXR; chest x-ray.
