# Supplementary material for: The association between FABP7 serum levels with survival and neurological complications in acetaminophen-induced acute liver failure: a nested case–control study
Source: Ann Intensive Care. 2017 Oct 5;7:99. doi: 10.1186/s13613-017-0323-0 (PMC5629189; doi:10.1186/s13613-017-0323-0)
Supplement: Supplementary file 4 — Additional file 4. Table S2. Biochemical and organ support parameters early (admission) and late (days 3–5) stratified cerebral edema. [file 13613_2017_323_MOESM4_ESM.docx]

**Table S2:** Biochemical and organ support parameters early (admission) and late (day 3-5) stratified cerebral edema.

| **EARLY (n=150)** | **APAP Cerebral Edema**  **(n=46)** | | **APAP No Cerebral Edema**  **(n=104)** | |  |
| --- | --- | --- | --- | --- | --- |
|  | N | Number (%) or median (IQR) | N | Number (%) or median (IQR) | P value |
| **Biochemistry** |  |  |  |  |  |
| Hemoglobin (g/dL) | 44 | 11.0 (9.2-12.3) | 104 | 10.7 (9.5-12.3) | 0.76 |
| White Blood count (10^9^/L) | 44 | 11.0 (6.9-18.9) | 104 | 9.3 (6.6-13.0) | 0.042 |
| Platelet count (10^9^/L) | 44 | 129.0 (66.0-195.0) | 104 | 109.0 (67.5-145.0) | 0.099 |
| INR | 45 | 3.6 (2.3-4.9) | 102 | 2.9 (2.0-3.8) | 0.024 |
| ALT (IU/L) | 46 | 4046 (2580-6662) | 103 | 3167 (1535-5676) | 0.074 |
| Bilirubin (mg/dL) | 46 | 4.7 (3.3-6.4) | 103 | 5.0 (3.5-7.3) | 0.28 |
| pH | 42 | 7.4 (7.4-7.5) | 90 | 7.4 (7.3-7.5) | 0.20 |
| Ammonia (venous) (μmol/L) | 19 | 140 (90-306) | 40 | 103 (70-150) | 0.12 |
| Creatinine (mg/dL) | 44 | 2.6 (1.0-3.4) | 104 | 2.2 (0.9-3.5) | 0.71 |
| Lactate (mmol/L) | 26 | 6.6 (3.4-10.5) | 74 | 5.8 (2.9-10.3) | 0.39 |
| Phosphate (mg/dL) | 36 | 2.4 (1.9-3.6) | 85 | 3.0 (1.9-4.5) | 0.25 |
| **MELD** | 45 | 29.0 (23.3-33.0) | 101 | 27.1 (17.6-31.8) | 0.12 |
| **High Coma Grade (3 or 4)** | 46 | 35 (76%) | 102 | 60 (59%) | 0.043 |
| **Organ support** |  |  |  |  |  |
| Mechanical ventilation | 46 | 39 (85%) | 104 | 68 (65%) | 0.019 |
| Vasopressors | 46 | 13 (28%) | 104 | 35 (34%) | 0.51 |
| Renal Replacement therapy | 46 | 13 (28%) | 104 | 24 (23%) | 0.50 |
| **FABP7 (ng/ml)** | 46 | 259.7 (90.7-562.2) | 104 | 228.2 (99.8-454.8) | 0.61 |
|  | | | | | |
| **LATE (n=138)** | **APAP Cerebral Edema**  **(n=37)** | | **APAP No Cerebral Edema**  **(n=101)** | |  |
|  | N | Number (%) or median (IQR) | N | Number (%) or median (IQR) | P value |
| **Biochemistry** |  |  |  |  |  |
| Hemoglobin (g/dL) | 33 | 10.1 (9.4-10.7) | 97 | 10.2 (9.2-11.2) | 0.90 |
| White Blood count (10^9^/L) | 34 | 10.1 (6.5-14.6) | 96 | 10.3 (6.1-14.6) | 0.96 |
| Platelet count (10^9^/L) | 34 | 70.5 (61.0-120.0) | 97 | 75.0 (51.0-131.0) | 0.81 |
| INR | 34 | 2.1 (1.5-3.4) | 89 | 1.9 (1.4-2.8) | 0.37 |
| ALT (IU/L) | 33 | 972 (542-1955) | 94 | 1103 (534-1891) | 0.94 |
| Bilirubin (mg/dL) | 32 | 8.5 (5.2-12.1) | 94 | 7.9 (4.7-13.5) | 0.88 |
| pH | 33 | 7.4 (7.3-7.4) | 71 | 7.4 (7.3-7.5) | 0.26 |
| Ammonia (venous) (μmol/L) | 12 | 63 (46-113) | 15 | 95 (76-126) | 0.33 |
| Creatinine (mg/dL) | 35 | 2.4 (1.3-4.0) | 95 | 1.6 (0.8-3.2) | 0.073 |
| Lactate (mmol/L) | 12 | 3.4 (1.9-4.7) | 41 | 3.2 (2.2-5.9) | 1.00 |
| Phosphate (mg/dL) | 15 | 3.0 (2.3-3.8) | 56 | 3.0 (2.4-4.1) | 0.71 |
| **MELD** | 32 | 28.5 (21.7-31.9) | 86 | 23.7 (11.1-31.6) | 0.094 |
| **High Coma Grade (3 or 4)*** | 36 | 36 (100%) | 77 | 58 (75%) | 0.0004 |
| **Organ support** |  |  |  |  |  |
| Mechanical ventilation | 37 | 35 (95%) | 101 | 64 (63%) | <0.0001 |
| Vasopressors | 37 | 18 (49%) | 101 | 32 (32%) | 0.066 |
| Renal Replacement therapy | 37 | 14 (38%) | 101 | 26 (26%) | 0.17 |
| **FABP7 (ng/ml)** | 37 | 223.8 (110.7-536.9) | 101 | 192.0 (64.0-389.7) | 0.19 |

N: frequency. IQR: interquartile range. INR: international normalized ratio. AST: aspartate aminotransferase. ALT: alanine aminotransferase. MELD: Model for End-stage Liver Disease. *Hepatic encephalopathy grade according to West-Haven criteria.
